# Birth-Related Perineal Trauma in Low- and Middle-Income Countries: A Systematic Review and Meta-analysis

**DOI:** 10.1007/s10995-019-02732-5

**Published:** 2019-03-26

**Authors:** Magda Aguiar, Amanda Farley, Lucy Hope, Adeela Amin, Pooja Shah, Semira Manaseki-Holland

**Affiliations:** 1 Institute of Applied Health Research, University of Birmingham, Birmingham, UK; 2 Institute of Metabolism and Systems Research, College of Medical and Dental Sciences, University of Birmingham, Birmingham, UK; 3 Present Address: Department of Nursing and Midwifery, Institute of Health & Society, University of Worcester, Worcester, UK; 4 Faculty of Pharmaceutical Sciences, University of British Columbia, Vancouver, BC Canada

**Keywords:** Episiotomy, OASI, Birth-related perineal trauma, Systematic review, LMICs

## Abstract

*Introduction* Birth-related perineal trauma (BPT) is a common consequence of vaginal births. When poorly managed, BPT can result in increased morbidity and mortality due to infections, haemorrhage, and incontinence. This review aims to collect data on rates of BPT in low- and middle-income countries (LMICs), through a systematic review and meta-analysis. *Methods* The following databases were searched: Medline, Embase, Latin American and Caribbean Health Sciences Literature (LILACs), and the World Health Organization (WHO) regional databases, from 2004 to 2016. Cross-sectional data on the proportion of vaginal births that resulted in episiotomy, second degree tears or obstetric anal sphincter injuries (OASI) were extracted from studies carried out in LMICs by two independent reviewers. Estimates were meta-analysed using a random effects model; results were presented by type of BPT, parity, and mode of birth. *Results* Of the 1182 citations reviewed, 74 studies providing data on 334,054 births in 41 countries were included. Five studies reported outcomes of births in the community. In LMICs, the overall rates of BPT were 46% (95% CI 36–55%), 24% (95% CI 17–32%), and 1.4% (95% CI 1.2–1.7%) for episiotomies, second degree tears, and OASI, respectively. Studies were highly heterogeneous with respect to study design and population. The overall reporting quality was inadequate. *Discussion* Compared to high-income settings, episiotomy rates are high in LMIC medical facilities. There is an urgent need to improve reporting of BPT in LMICs particularly with regards to births taking in community settings.

**Electronic supplementary material:**

The online version of this article (10.1007/s10995-019-02732-5) contains supplementary material, which is available to authorized users.

## Significance

*What is already known on this subject?* Birth-related perineal trauma is a common complication of vaginal childbirth. Adequate management reduces morbidity and improves maternal health and wellbeing. Women in LMICs are thought to be at a higher risk of perineal trauma. *What this study adds?* Women in LMICs are at higher risk for episiotomy, but not for the spontaneous second degree tear and OASI. Better reporting practices and more evidence from the community are needed to provide a more accurate picture of the burden of BPT in LMICs.

## Introduction

Maternal health is critical in many countries of the world, and the World Health Organization (WHO 2018) estimates that 99% of maternal deaths happen in low- and middle-income countries (LMICs). However, death is only the “tip of the iceberg” that surfaces a devastating plethora of conditions affecting the health and wellbeing of mothers living in resource-poor settings. A report from the Safe Motherhood initiative, a partnership between the WHO, the World Bank, and other international organisations that aims to improve maternal and new-borns’ health in LMICs, estimated that for every mother who dies, 30–50 women suffer injury, infection, or disease (Islam 2007). While the burden of BPT in LMICs is not known, studies in high-income settings show that the majority of women who have a vaginal birth experience some form of BPT (Christine Kettle et al. 2012; Chris; Kettle and Tohill 2008). BPT refers to any injury to the perineum that happens during childbirth. BPT can happen as a spontaneous tear due to pressure on the perineum when the baby is delivered vaginally, or as a surgical cut, known as episiotomy, that aims facilitate vaginal birth and prevent severe spontaneous tears (Carroli and Mignini 2009; Royal College of Midwives 2012). Spontaneous BPT is classified as a first degree tear if there is only injury to the skin; second degree tear if there is injury to the skin and the muscle tissue; and obstetric anal sphincter injuries (OASI), which include third and four degree tears, if the injury extends to the anal sphincter (Chris Kettle and Tohill 2008). The main risk factors for BPT include maternal age, parity, use of forceps, birthweight, and prolonged second stage labour (Smith et al. 2013). Inadequate management of BPT can lead to severe complications. Acute complications include haemorrhage and puerperal sepsis, which are major causes of death in LMIC, while chronic complications include pelvic floor disorders, such as urinary and faecal incontinence (Huebner et al. 2013; Poen et al. 1998) persistent pain, dyspareunia, and prolapse (Elharmeel et al. 2011; WHO 2003).

Episiotomy and OASI rates are commonly used as quality indicators of health systems and health care (WHO 2016). Misdiagnose and underreporting of perineal tears have been cited as the main barriers to the improvement of BPT management and morbidity-related outcomes in Europe (Blondel et al. 2016). The same is likely to occur in LMICs, with even more devastating impact on health and wellbeing. Young women suffering from chronic incontinence and dyspareunia suffer from lower quality of life and self-esteem (Sinclair and Ramsay 2011). Socially, these conditions create a hostile environment stigma, isolation and rejection by the husband and the community, in turn leading to emotional burden and shame (Mota 2017). Although the actual numbers are not known, it is speculated that BPT affects millions per year around the world, who suffer with its consequences in silence (WHO 2009).

The high rate of community deliveries by untrained birth attendants (UNICEF 2008), the young maternal age at first pregnancy, and the high rates of episiotomies in hospitals, led us to hypothesise that women in LMICs are at a higher risk of BPT. The risk of complications associated with BPT is also likely to be increased in poorer settings due to the limited access to the adequate resources such as optimal suturing materials, poor environmental and household circumstances, lack of sanitation, and malnutrition (UNICEF 2008). In view of the above, there is an urgent need to understand the scale and characteristics of the problem in LMICs. Hence, the aim of this systematic review was to summarise data on the use of episiotomy and the frequency of spontaneous significant BPT (second degree and OASI) in LMICs.

## Methods

This review follows the Preferred Reporting Items for Systematic Reviews and Meta-Analyses (PRISMA) and the Meta-Analysis of Observational Studies in Epidemiology (MOOSE) (Moher et al. 2010; Stroup et al. 2000). The protocol has been published elsewhere (Aguiar et al. 2013). Searches were conducted in the following electronic databases: Embase (1996–2016); Medline, (1996–2016), Lilacs and the WHO’s regional databases (African Index Medicus, Index Medicus for the Eastern Mediterranean Region, Index Medicus for South-east Asian Region and Western Pacific Region Index Medicus), and took place in February/March 2014 and updated in February 2016. Search strategies (supplementary material) were constructed by combining MeSH terms and key words relating to the perineum, childbirth, episiotomy, and low and middle income countries. We also searched reference lists of the included studies and performed google searches to identify unpublished work.

### Study Eligibility

Studies were included in the review if they (1) reported data on the proportion of vaginal births resulting in perineal trauma (episiotomy, second degree trauma and OASI); (2) were conducted in a LMIC as defined by the World Bank (2014); (3) reported data from 2004 to 2016 (this time-frame was chosen to maximize the relevance for current clinical practice and policy recommendations). All study designs were considered, as long as cross-sectional data were available. In interventional studies, data were retrieved from the usual care arm.

### Study Identification and Data Extraction

Eligible studies were identified by a two stage screening process. Firstly, two reviewers independently screened titles and abstracts (MA and LH) and secondly, the full texts of the potentially relevant studies were screened (MA, AA and PS). Data were extracted independently and in duplicate using a predesigned data extraction form (see supplementary material). We extracted data regarding the study setting and design, recruitment method, parity, and characteristics of included participants. Any discrepancies during screening or data extraction were resolved through discussion or through the input of a clinical reviewer (SMH) if consensus could not be reached.

### Quality Assessment

The quality of included studies was assessed by two reviewers (MA and PS) using a bespoke tool based on Looney’s critical appraisal instrument for systematic reviews of incidence and prevalence studies (Loney et al. 1998) and the WHO systematic review of severe acute maternal morbidity (Gülmezoglu et al. 2004). Quality was based on definition of perineal trauma, sample size, loss of data, and adequacy of description of the population’s characteristics (supplementary material).

### Statistical Analysis

Estimates of the proportion of vaginal births that resulted in BPT were pooled by the different types of BPT (episiotomy, second degree tears and OASI) using a random effects model. Random effects was chosen due to expected high clinical and statistical heterogeneity (determined by *I*^2^ statistic > 75%). (Nyaga et al. 2014). Sub-group analyses by parity and delivery mode (spontaneous or operative vaginal birth) were also performed and presented in supplementing material.

## Results

### Identification of Studies

**Fig. 1:**
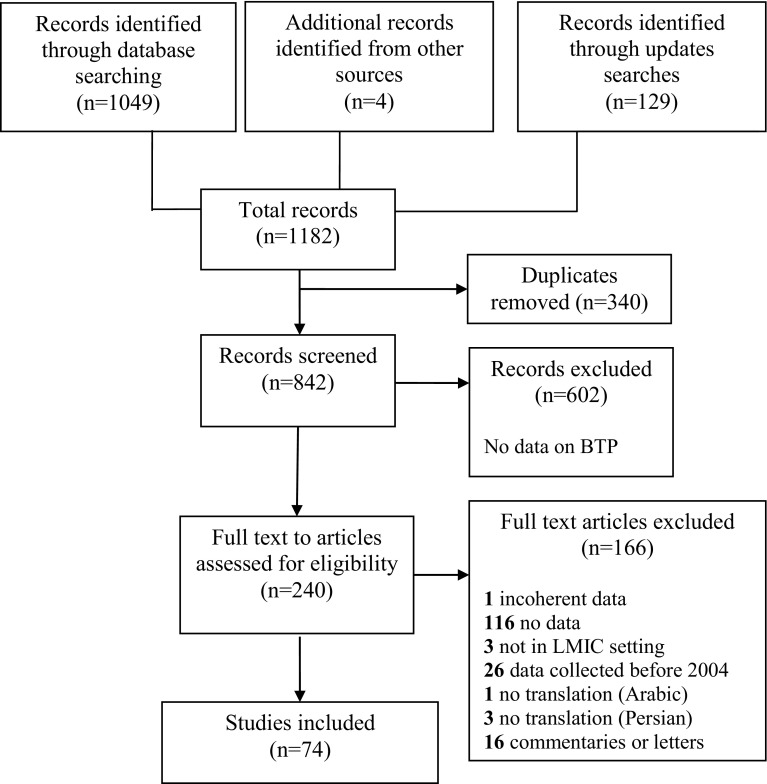
Flow diagram of the study selection process

A total of 1182 studies were identified. After duplicates were removed (n = 340), the remaining 842 articles were screened for eligibility based on their title and abstract. The full text of 240 studies were assessed and of these, 74 studies were included (Fig. 1).

A summary of characteristics of included studies is provided in Table 1. This review provides data from 41 LMICs, the majority being lower-middle (*n* = 19) and upper-middle income (*n* = 16) countries. Studies varied in their designs with most being cross-sectional (*n* = 27). There were also cohort studies (*n* = 20), RCTs (*n* = 15), and non-randomised interventional studies (*n* = 6). Several studies provided data on more than one type of BPT. Overall, this review found 118 BPT estimates, of which 46 were episiotomies, 13 s degree trauma, 42 OASI, and 16 were not specified (NS).

**Table 1:** Included study characteristics

Data overview	
Total number of studies	74
Total number of vaginal births	334,054
Total number of countries	42
Countries’ income status^a^	
Low income	7
Lower-middle income	19
Upper-middle income	16
Study design	
Cross-sectional	28
Cohort	12
RCT	15
Other	19
Place of birth	
Medical facility	69
Mixed	3
Home	2
Type of delivery^a^	
Assisted delivery	11
Spontaneous delivery	14
Mixed	47
Not stated	46
Type of trauma^a^	
Episiotomy	46
2nd degree	14
OASIS	42
Not defined	16
Parity^a^	
Primiparous	31
Multiparous	3
Mixed	68
Not stated	16

^a^The number of studies in each category will might not add to 74, as several studies might reported more than one tpe BPT

The majority of the studies reported episiotomy data (*n* = 46) and were set in medical facilities (*n* = 69). Three studies reported combined results of births in medical facilities and in the community (Assarag et al. 2013; Gözükara et al. 2015; Iyengar 2012) and one study, by Koettker et al. (2012), focus on planned home births with women with low risk pregnancies. Most studies’ population were healthy primiparous women with singleton pregnancies. Several studies (*N* = 68) included women of various parities. Those studies that did not stratify the outcome by parity were classified as having a mixed-parity population. The main characteristics of each of the included studies are provided in Table 2. A few studies focused on specific sub-groups of the population: breastfeeding mothers, (Nguyen et al. 2013) multiparous women, (Reyes 2011) women subjected to female genital mutilation (Kaplan et al. 2013; Ndiaye et al. 2010), prolonged second stage of labour (Colacioppo and Gonzalez Riesco 2009) and birth of a macrocosmic foetus (Chaabane et al. 2013). Others focused on different birth techniques or settings: operative vaginal births, (Baloch et al. 2008; Carvalho et al. 2010; Khaskheli et al. 2012; Waheed et al. 2012) supine birthing position (Aguilar et al. 2013) and planned home birth for low risk pregnancies (Koettker et al. 2012).

**Table 2:** Main characteristics of included studies

Study	Country (study period)	Study design	Place of birth	Case identification	Parity	Type of delivery	*N* (Sample size)	Episiotomy (%)	2nd degree tear (%)	OASI (%)	NS (%)
Singh and Rathore (2011)	India (August 2007 to February 2009)	Cross-sectional	Medical facility (hospital)	Consecutive sample of operative delivery from medical records (vacuum and forceps)	NS	Operative	120	86.7			
Calvo et al. (2013)	Mexico (March 2011 to April 2012)	RCT (Cross-sectional data)	Medical facility (hospital)	Low risk pregnancies with singleton pregnancy on cephalic presentation	Mixed	Normal	78		12.8	2.6	
Alayande et al. (2012)	Nigeria (September 2008 to February 2005)	Cross-sectional	Medical facility (hospital)	All vaginal deliveries during study period, based on medical records	Primiparas	Normal	280	34.3			
Francisco et al. (2011)	Brazil (2007)	Cross-sectional	Medical facility (hospital)	Convenience sample of singleton pregnancies. Women > 15 years	Mixed	Mixed	303	60.7	6.6		80.5
Assarag et al. (2013)	Morocco (December 2010 to March 2012)	Cross-sectional	Mixed	Whole population (all women giving birth during study period). Limited to women aged between 18 and 49 years old	Mixed	NS	1530	34.5			
Baloch et al. (2008)	Pakistan (2005–2006)	Cross-sectional	Medical facility (hospital)	Consecutive sample of vacuum delivery	Mixed	Operative	160			2.5	17.5
Bello et al. (2010)	Nigeria (August 2008)	Cross-sectional	Medical facility (hospital)	All vaginal deliveries during study period	Multiparas	NS	375	25.1			
Braga et al. (2014)	Brazil (March 2009 - July 2010)	Case-control (cross sectional data)	Medical facility (hospital)	Convenience sample of assisted vaginal deliveries	Mixed	Mixed	522	33.1			
Brohi et al. (2012)	Pakistan (December 2009–May 2010)	Case series (Cross-sectional data)	Medical facility (hospital)	Deliveries of singletons in cephalic presentation	Primiparas	NS	1488		3.2	3.8	
Cawich et al. (2008)	Jamaica (2004–2006)	Retrospective case series (Cross sectional data)	Medical facility (hospital)	All vaginal deliveries during study period	Mixed	NS	3957			0.2	
Chang et al. (2007)	Ecuador (August 2004–January 2005)	Case series (Cross sectional data)	Medical facility (hospital)	Singleton pregnancy with cephalic presentation subjected to vacuum delivery	NS	Operative	97	74.2	5.2		11.3
Wai et al. (2015)	China (January 2011–June 2014)	Cohort	Medical facility (Obstetrics and Gynaecology Centre)	All vaginal deliveries of a singleton baby during study period	Mixed	Mixed	15,526			3.2	
Colacioppo and Riesco (2009)	Brazil (June 2004–December 2004)	RCT (Cross-sectional data)	Medical facility (Birth centre)	Healthy women with singleton pregnancy on cephalic presentation. Age > 15 years old	Primiparas	NS	105	30.5	31.4		
Colacioppo et al. (2011)	Brazil (January 2009–June 2009)	RCT (Cross-sectional data)	Medical facility	Healthy women with singleton pregnancy on cephalic presentation	Primiparas	NS	77	10.4	24.7	5.2	
Conde-Agudelo et al. (2008)	Colombia (April–December 2006)	Mixed methods	Medical facility (hospital)	All low-risk pregnancies during study period	Primiparas	NS	1410	79.8			
da Silva et al. (2012)	Brazil (January 2006–December 2009)	Cross-sectional	Medical facility (Birth centre)	All vaginal deliveries during study period	Primiparas	NS	1079	32.2	23.6		
Gómez Dávila et al. (2006)	Colombia (July 2004–April 2006)	Cohort	Medical facilities (1 public hospital and 2 private)	All vaginal deliveries with 24 weeks gestational age, during study period	Primiparas	NS	669	71.4			
Carvalho et al. (2010)	Brazil (January–December 2006)	Retrospective study (Cross-sectional data)	Medical facility (hospital)	Random sample of whole population	Primiparas	NS	495	29.1			
Demirel and Golbasi (2015)	Turkey (January 2010 and May 2011)	RCT (Cross-sectional data)	Medical facility (hospital)	Healthy women or second birth, cephalic presentation, gestational age of 37–42 weeks of pregnancy, and in the latent phase of the first stage of labour with dilatation of less than 4 cm and effacement of less than 50%	Mixed	Normal	142	42.2			8.4
Egbe et al. (2015)	Cameroon (2009–2012)	Cross-sectional	Medical facility (1 hospital and 2 health centres)	Healthy women with singleton pregnancy and gestational age > 28 weeks. Uncomplicated births. Age 14–29 years old	Primiparas	NS	148	3.3			18.9
Farooq et al. (2010)	Pakistan (2004)	Cohort	Medical facility (hospital)	All deliveries	Mixed		2563				10
Ferdous et al. (2012)	Bangladesh (2007–2008)	Cohort	Mixed (medical facility and community)	Random sample of the whole population	Mixed	NS	482		1.1	0.04	10.1
Figueiredo et al. (2011)	Brazil (2008)	Cross-sectional	Medical facility (hospital)	All dystocia-free vaginal deliveries assisted by nurses	Mixed	NS	447	11.2			
Azam Foroughipour et al. (2011)	Iran (2007–2008)	RCT (Cross-sectional data)	Medical facility (hospital)	For this review only the arm that represented the common practice, the hand group, was considered. Age 15–35 years old	Primiparas	Normal	50	80	26		
Fouelifack et al. (2014)	Cameroon (2008 and 2010)	Intervention study (Cross-sectional data)	Medical facility (hospital)	All vaginal deliveries	Mixed	Mixed	5045	6.7	0.4	< 0.1	
Frass and Al-Harazi (2010)	Yemen (2008)	Cohort	Medical facility	Whole population (all women giving birth during study period)	Primiparas	NS	2588	75.1		0.27	
Fyneface-Ogan et al. (2006)	Nigeria (January to December 2008)	RCT (Cross-sectional data)	Medical facility (hospital)	Data for this review was extracted from repost of records of all vaginal births	Mixed	Normal	1293	37.3			1.4
Garcia-Elorrio et al. (2014)	Nicaragua (August 2011 and April 2012)	Intervention study (Cross-sectional data)	Medical facility (several medical facilities)	All vaginal deliveries	Mixed	Normal	766	31.2	2.7	0.3	
Geranmayeh et al. (2012)	Iran (2009)	RCT (Cross-sectional data)	Medical facility (hospital)	All vaginal deliveries with baby in cephalic presentation. Limited to women aged between 18 and 30 years old	Mixed	Normal	45	84.4	2.2		
Gözükara et al. (2015)	Turkey (March–April 2013)	Cross-sectional	Mixed (home and medical facilities at regional level)	All vaginal deliveries	NS	NS	279	99.2			
Hafeez et al. (2013)	Pakistan (2010–2011)	Case series	Medical facility	Women undergoing vacuum assisted delivery	Mixed	Operative	67			1.49	
Hasegawa and Leventhal (2009)	Brazil (2008)	Cohort	Medical facility (hospital)	All deliveries	Mixed	Mixed	130				97.8
Hassan et al. (2013)	Occupied Palestinian Territory (2005–2006)	Mixed methods (Cross-sectional data)	Medical facility	Interviews with midwives and physicians regarding their practice	Primiparas	Mixed	134	79.9			
Hirayama et al. (2012)	Algeria (2004–2005)	Cross-sectional	Medical facility	All vaginal deliveries	Mixed	Mixed	13,654			7.2	
	Angola (2004–2005)	Cross-sectional	Medical facility	All vaginal deliveries	Mixed	Mixed	6298			0.7	
	DR Congo (2004–2005)	Cross-sectional	Medical facility	All vaginal deliveries	Mixed	Mixed	7894			0.9	
	Kenya (2004–2005)	Cross-sectional	Medical facility	All vaginal deliveries	Mixed	Mixed	17,063			1.1	
	Niger (2004–2005)	Cross-sectional	Medical facility	All vaginal deliveries	Mixed	Mixed	7976			2.8	
	Nigeria (2004–2005)	Cross-sectional	Medical facility	All vaginal deliveries	Mixed	Mixed	7813			1.4	
	Uganda (2004–2005)	Cross-sectional	Medical facility	All vaginal deliveries	Mixed	Mixed	12,135			0.6	
	Cambodia (2004–2005)	Cross-sectional	Medical facility	All vaginal deliveries	Mixed	Mixed	4812			0.1	
	China (2004–2005)	Cross-sectional	Medical facility	All vaginal deliveries	Mixed	Mixed	7867			0.1	
	India (2004–2005)	Cross-sectional	Medical facility	All vaginal deliveries	Mixed	Mixed	20,519			0.1	
	Nepal (2004–2005)	Cross-sectional	Medical facility	All vaginal deliveries	Mixed	Mixed	6817			0.5	
	Philippines (2004–2005)	Cross-sectional	Medical facility	All vaginal deliveries	Mixed	Mixed	10,879			15	
	Sri Lanka 2007–2008	Cross-sectional	Medical facility	All vaginal deliveries	Mixed	Mixed	10,494			0.4	
	Thailand (2007–2008)	Cross-sectional	Medical facility	All vaginal deliveries	Mixed	Mixed	6454			0.9	
	Vietnam (2007–2008)	Cross-sectional	Medical facility	All vaginal deliveries	Mixed	Mixed	8607			0.3	
	Argentina (2004–2005)	Cross-sectional	Medical facility	All vaginal deliveries	Mixed	Mixed	6753			0.3	
	Brazil (2004–2005)	Cross-sectional	Medical facility	All vaginal deliveries	Mixed	Mixed	10,720			0.7	
	Cuba (2004–2005)	Cross-sectional	Medical facility	All vaginal deliveries	Mixed	Mixed	8195			0.4	
	Ecuador (2004–2005)	Cross-sectional	Medical facility	All vaginal deliveries	Mixed	Mixed	7437			2.2	
	Mexico (2004–2005)	Cross-sectional	Medical facility	All vaginal deliveries	Mixed	Mixed	13,028			0.8	
	Nicaragua (2004–2005)	Cross-sectional	Medical facility	All vaginal deliveries	Mixed	Mixed	3912			0.4	
	Paraguay (2004–2005)	Cross-sectional	Medical facility	All vaginal deliveries	Mixed	Mixed	2024			1.6	
	Peru (2004–2005)	Cross-sectional	Medical facility	All vaginal deliveries	Mixed	Mixed	10,655			0.3	
Ho et al. (2010)	Indonesia (2005)	Before and after study (Cross-sectional data)	Multi country	All vaginal deliveries during study period	NS	NS	1146	53.5			
	Malaysia (2005)	Before and after study (Cross-sectional data)	National (multi country)	All vaginal deliveries during study period	NS	NS	1700	60.6			
	Philippines (2005)	Before and after study (Cross-sectional data)	National (multi country)	All vaginal deliveries during study period	NS	NS	1274	63.7			
	Thailand (2005)	Before and after study (Cross-sectional data)	National (multi country)	All vaginal deliveries during study period	NS	NS	1568	91.6			
Inyang-Etoh and Umoiyoho (2012)	Nigeria (12months, month and year not reported)	Cross-sectional	Medical facility (hospital)	All vaginal deliveries during study period	NS	NS	1306	21.1			
Iyengar (2012)	India (January 2007–Dec 2010)	Intervention study (Cross-sectional data)	Mixed (Home and health centres)	All women receiving nurse/midwives home-level postnatal visits	NS	NS	1156	7.1			1.2
Joshi and Acharya (2009)	Nepal (August 2005–November 2005)	cohort	Medical facility (hospital)	All low risk with singleton pregnancy on cephalic presentation and gestational age between 36 and 42 weeks	Primiparas	NS	410	22.2			
Kaplan et al. (2013)	Gambia (December 2010–March 2011)	Cohort	Medical facilities	All vaginal deliveries during study period	Mixed	Mixed	381	16			27.8
Karaçam et al. (2012)	Turkey (2006–2009)	RCT	Medical facility (hospital)	All singleton pregnancies. Limited to women between 18 and 35 years old	Primiparas	Normal	10,050	60.6	3.5	15.7	96.5
Khaskheli et al. (2012)	Pakistan (2006–2010)	Case series (Cross sectional data)	Medical facility (hospital)	All cases admitted in maternity ward. The study included all the women delivering in the hospital and all referred from outside either from local maternity homes, rural health centres and delivery at home within 40 days	Mixed	NS	9216				0.6
Khresheh et al. (2009)	Jordan (July–August 2004)	Cross-sectional	Medical facility (hospital)	All vaginal deliveries during study period	Mixed	Mixed	905	54.3		0.45	15.8
Koettker et al. (2012)	Brazil 2005–2009	Cross-sectional	Home	All women with planned home birth (low risk pregnancies)	Mixed	NS	89	1.1			
Kongnyuy et al. (2008)	Cameroon (2004–2005)	Cross-sectional	Medical facility (hospital)	Singleton pregnancy	Primiparas	Mixed	1003	11.6			
Saxena et al. (2010)	India (2007)	Cohort	Medical facility (hospital)	All vaginal deliveries during study period	Primiparas	Mixed	210	67.6	0.95		
Moghadam et al. (2015)	Iran	Cross-sectional	Medical facility (multiple health centres)	All vaginal deliveries during study period	NS	Mixed	400	36			
Moini et al. (2009)	Iran	RCT (Cross-sectional data)	Medical facility (hospital)	Whole population (all women giving birth during study period)	Primiparas	NS	146	51.2		7.4	
Mola and Kuk (2010)	Papua New Guinea (2007)	RCT (Cross-sectional data)	Medical facility (hospital)	Singleton pregnancy with cephalic presentation subjected to vacuum delivery	Mixed	Operative	100	60		1	
Mollamahmutoğlu et al. (2012)	Turkey (June 2007–September 2008)	RCT (Cross-sectional data)	Medical facility (hospital)	Convenience sample of low risk pregnancies	Primiparas	NS	204	89.2			1.5
Ndiaye et al. (2010)	Burkina Faso (June–August 2007)	Cross-sectional	Medical facility (4 maternity hospitals)	All vaginal deliveries during study period	NS	NS	330	23.6			4.5
Nguyen et al. (2013)	Vietnam (2011)	Cross-sectional	National	Based on household survey. Cluster sample of pairs of breastfeeding mothers and children under 24 months	NS	NS	6068	41.3			
Nkwabong et al. (2009)	Cameroon (May to October 2010)	Case series	Medical facility (hospital)	Singleton pregnancy with cephalic presentation	Mixed	NS	1695	9.6		0.18	86.4
Njoku et al. (2015)	Nigeria (January 2009–December 2014)	Case series	Medical facility (hospital)	All vaginal deliveries during study period	NS	NS	15,526				9
Obioha et al. (2015)	Nigeria (November 2012–June 2013)	Cross-sectional	Medical facility (hospital)	All vaginal deliveries during study period	Mixed	Mixed	208	38			6.6
Oliveira et al. (2014)	Brazil (2009–2010)	Cross-sectional	Medical facility (hospital)	Vaginal delivery, gestational age > 37 weeks, cephalic presentation	Primiparas	Mixed	1384		25	1.1	
Rathfisch et al. (2010)	Turkey 92,005-2006	Descriptive prospective (Cross-sectional data)	Medical facility (hospital)	All low-risk pregnant women who expected vaginal delivery at over 38-weeks of gestation with a single foetus in the vertex position	Primiparas	NS	165	67.3			
Reyes (2011)	Panamá (2007–2009)	Cohort	Medical facility	Whole population (all women giving birth during study period)	Mixed	Mixed	3573	21.6			
Rojas-Higuera et al. (2006)	Colombia (Aug 2004–March 2005)	Cross-sectional	Medical facility (hospital)	All vaginal deliveries with gestational age > 24 weeks	NS	Mixed	906	56.5			
Sagili et al. (2012)	India (March 2008–April 2009)	Cross-sectional	Medical facility (hospital)	All vaginal deliveries during study period, based on medical records	Mixed	Mixed	15,498	52.58			8.3
Saleem et al. (2016)	Pakistan (January–June 2008)	Case series (Cross-sectional data)	Medical facility (hospital)	Consecutive sample of operative delivery (vacuum and forceps)	Primiparas	Operative	120		7.5	0.05	
Salge et al. (2012)	Brazil (2009–2010)	Cross-sectional	Medical facility (maternity hospitals)	All vaginal deliveries during study period, based on medical records	Primiparas	NS	1129	56.3			
de Oliveira Santos et al. (2012)	Brazil (2009)	RCT (Cross-sectional data)	Medical facility (hospital)	All vaginal deliveries during study period, based on medical records	Mixed	NS	1872	18.8			40.9
Santos et al. (2008)	Brazil (July to December 2006)	Cross-sectional	Medical facility (hospital)	All women giving birth during study period	NS	NS	246	90.2			3.3
Shahraki et al. (2011)	Iran (October 2007–September 2008)	Cross-sectional	Medical facility (hospital)	Healthy women	NS	NS	36		17.5	2.5	
Shirvani and Ganji (2014)	Iran (September 2011–March 2012)	RCT (Cross-sectional data)	Medical facility (2 hospitals)	Healthy women, age of 18–35, gestational age of 37–41 weeks, single pregnancy, cephalic presentation and cervix dilatation of 3–4 cm	Primiparas	NS	32	94			
Sooklim et al. (2007)	Thailand (April 2005–February 2006)	Prospective cohort	Medical facility (hospital)	Deliveries of singletons in cephalic presentation. All received episiotomy	Primiparas	NS	1302			9.5	
Sorensen et al. (2011)	Tanzania (2008)	Prospective intervention study	Medical facility (hospital)	Not clear	Mixed	NS	510	18.4			
Spitzer et al. (2014)	Kenya (August–November 2011)	Prospective cohort	Medical facility (hospital)	All vaginal deliveries during study period	Mixed	Mixed	1451	13.2	2.5	0.6	
Sulaiman et al. (2013)	Malaysia (2009)	RCT (Cross-sectional data)	Medical facility (hospital)	Healthy women with singleton pregnancy on cephalic presentation	Primiparas	NS	82		96.3	3.7	
Vogt et al. (2011)	Brazil (2006)	Cross-sectional	Medical facility (3 hospital)	All low risk pregnancies	Mixed	NS	277	7.2			
Waheed et al. (2012)	Pakistan (2009–2010)	Quasi experimental	Medical Facility	Singleton pregnancy subjected to operative delivery. Women aged between 20 and 40 years	NS	Operative	60			1.7	58.3
Wang et al. (2014)	China (December 2010–March 2011)	RCT (Cross-sectional data)	Medical Facility (3 hospitals)	Healthy women, singleton, 16–32 weeks of gestation	Primiparas	Normal	28	47.1			7.8
Yadav et al. (2008)	Nepal (July 2005–June 2006)	Cohort	Medical facility (hospital)	Whole population (all women giving birth during study period)	Mixed	Normal	4101	12.6			
Zhu et al. (2012)	China (September 2007–May 2009)	Cross-sectional	National	All women giving birth during study period	Mixed	Mixed	5013	69.7			21

*NS* not stated

**Fig. 2:**
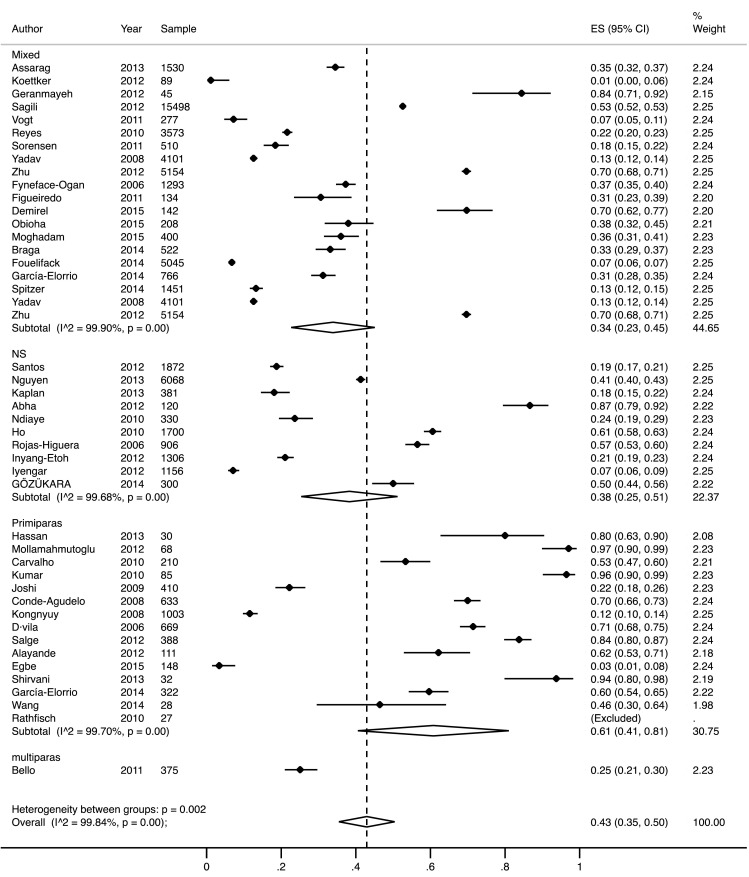
Forest plot showing results from meta-analysis of the frequency of episiotomy. *NS* parity not stated in the study

Overall, the meta-analysis estimated that 46% (95% CI 35–55%) of vaginal births in LMICs were facilitated by an episiotomy (Fig. 2). A sub-group analysis by parity showed that primiparous women were at higher risk for episiotomy, 62% (95% CI 40–84%), compared to mixed parity populations 33% (95% CI 22–45%), and multiparous population 25% (CI 21–30%). The overwhelming majority of these births happened in medical facilities.

**Fig. 3:**
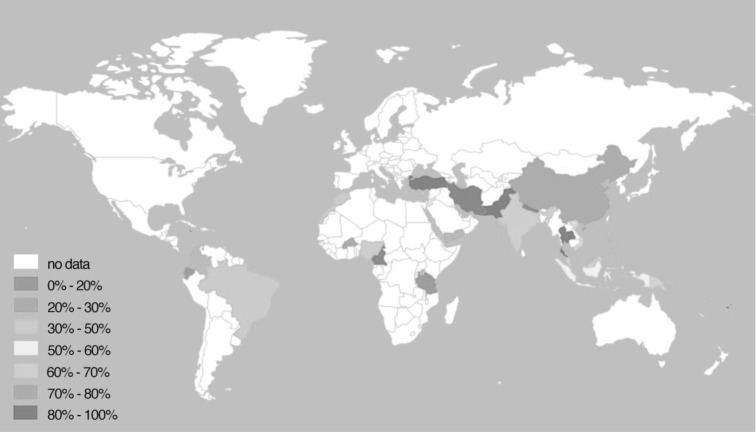
World map showing the frequency of episiotomy by country

A representation of the meta-analysis results by country (Fig. 3) shows that reported rates of episiotomy were generally higher in the Asian continent, nevertheless, data were lacking for the majority of LMICs. The highest pooled estimates were in Pakistan, with 98% (CI 93–99%), and the lowest in Cameroon, with 10% (CI 9–11%).

**Fig. 4:**
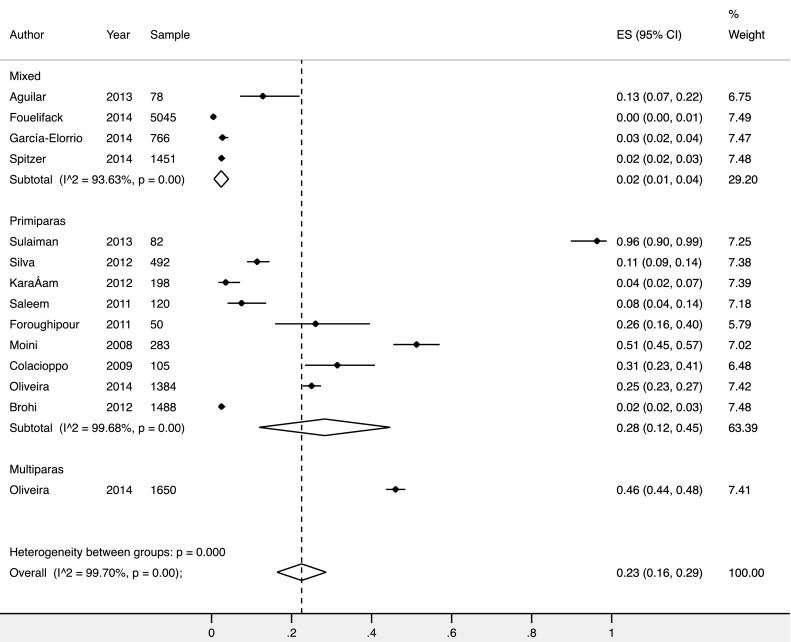
Forest plot showing results from meta-analysis of the frequency of second degree trauma by parity. *NS* parity not stated in the study

The overall reported rate of spontaneous second degree tears was 23% (95% CI 16–29%) (Fig. 3). The frequency of second degree tears was higher in studies reporting on primiparous women, 32% (95% CI 11–52%), compared to mixed parity populations, 3% (95% CI 1–4%) (Fig. 4).

**Fig. 5:**
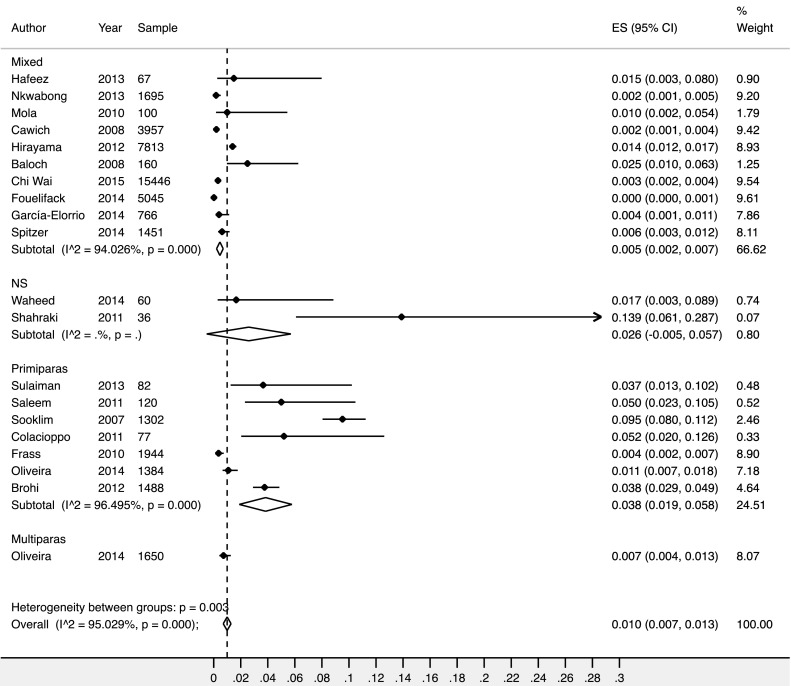
Forest plot showing results from meta-analysis of the frequency of oasis by parity. NS – parity not stated in the study

Following a similar trend, OASI occurred more frequently in primiparous women, 3.9% (95% CI 1.7–6%)—than in mixed parity populations, 1.4% (95% CI 1.1–1.6%). The overall reported OASI rate was 1.4% (95% CI 1.2–1.7%) (Fig. 5).

**Fig. 6:**
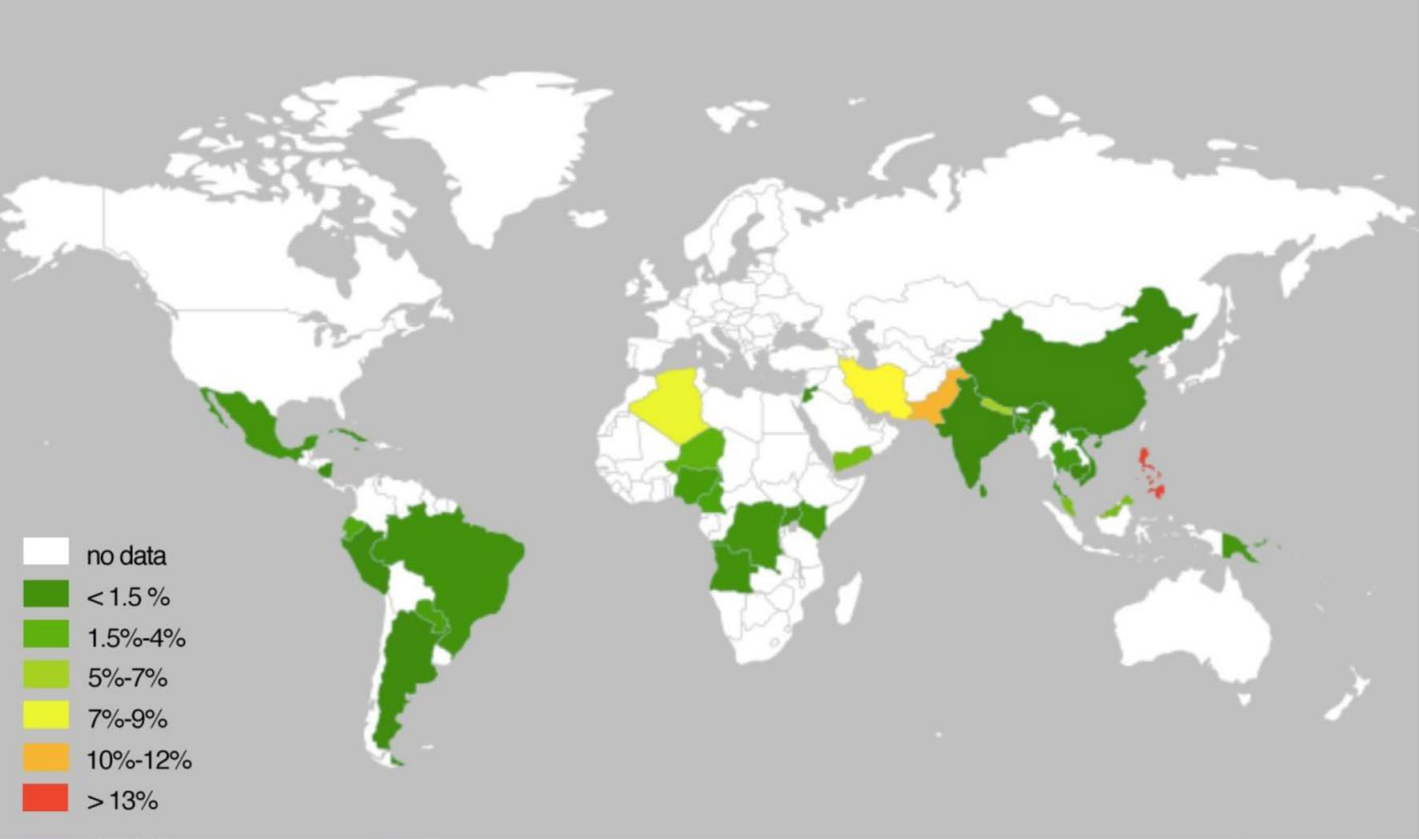
World map showing the frequency of oasis by country

A geographic representation of the pooled rates of OASI by country (Fig. 6) shows that, similar to episiotomy rates, data were not available for most of LMICs. Philipines had the highest reported rate of 15% (CI 14–16%), followed by 10% (CI 3–17%) for Pakistan. The lowest pooled reported rate in the meta-analysis was 0.1% (CI 0.04–0.2%), in Cambodia. Further meta-analyses estimating the frequency of BTP by mode of delivery were undertaken and are presented in the supplementary material.

### Quality Assessment

**Fig. 7:**
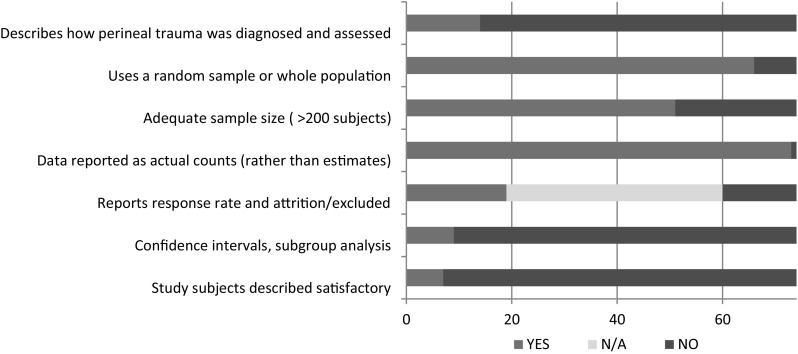
Quality assessement results

The quality assessment revealed poor reporting standards, with only a minority of the studies providing satisfactory description of how perineal trauma was defined or the characteristics of the studied population (Fig. 5). There was a general effort to avoid selection bias by attempts to include all eligible individuals, and in most studies, the breakdown of the results was reported with the crude estimates rather than summary statistics (Fig. 7).

## Discussion

This systematic review collected data from over 300,000 vaginal births in LMICs to estimate the frequency of episiotomy, second degree tears and OASI. Overall, we estimated that 46% (95% CI 35–55%) of the vaginal births in LMICs were facilitated by an episiotomy, while 23% (95% CI 16–29%) resulted in a spontaneous second degree tear and 1.4% (95% CI 1.2–1.7%) in OASI.

The use of episiotomy is controversial—while there is no evidence to support the use of routine episiotomy to protect from severe perineal tears (Jiang et al. 2017), a recent systematic review shows that women receiving mediolateral episiotomy are less likely to suffer from a severe BPT (Verghese et al. 2016). Selective episiotomy practices are recommended and the WHO states that fewer than 10% of vaginal births should receive an episiotomy (WHO 1997). Nonetheless, routine use of episiotomy is still common (Lowenstein et al. 2005). In light with these, our results showed that the chance of having an episiotomy is high in births happening in medical facilities, and primiparous women are at a higher risk for all types of BPT. Regarding OASI, our results did not show that women in LMICs are at a higher risk, compared to the average rate of 1.6% in the Organisation for Economic Co-operation and Development (OECD) countries (OECD 2011). It is likely that the lower rate of OASI we have found in this review is linked to barriers to data collection and underreporting issues in LMICs settings. This is an important issue that needs further investigation since OASI is an important cause of morbidity in LMICs and the incontinence and impaired sexual function resulting from OASI might affect marital relationships, reduce productivity and lead to social isolation in LMIC (WHO & United Nations Population Fund 2009).

Women in LMICs are often advised to give birth in medical facilities (Goldenberg and McClure 2017; Roro et al. 2014) since these are considered safer environments. Nonetheless, our findings suggest that routine episiotomy is widely used in some medical practices, raising concerns regarding the quality of the care women receive in these settings. Despite efforts to increase access to medical facilities in LMICs, the proportion of births that take place in the community is high (UNICEF 2008). It has been estimated that 60 million births occur in the community (Darmstadt 2009), where access to health care facilities is compromised for many but there are strongly rooted community-based health care systems. Nonetheless, the majority of the included studies reported births in medical facilities, denoting a dearth of data on community births. Childbirth happening outside medical facilities, with restricted resources might mean that serious birth-related complications will be more dangerous to the mother and the child (Pasha et al. 2013; Roro et al. 2014). The lack of data on community births was somehow expected. Collection of routine data requires an appropriate structure and trained community workers that poor resource settings lack. Even when such structures are in place, the outcomes of interest are more likely to be maternal and infant mortality related than complications that are perceived as being less severe. Underreporting has been shown to be a problem for outcomes of childbirth in LMICs (Målqvist 2008) but the evidence shows that training community health workers on data collection covering successfully impact the quantity and quality of available data in Ethiopia, Malawi and Mali (Silva et al. 2016). Ideally, data on BPT would be collected alongside vital outcomes, in acknowledgement of its high impact on woman’s health and wellbeing.

The results of the meta-analyses show high heterogeneity and wide variation within settings, with studies reporting episiotomy rates ranging from 1% (Koettker et al. 2012) in a Brazilian study of planned home births in low-risk pregnancies, to as high as 99%, as reported by Gözükara et al. in several medical facilities in Thailand (Gözükara et al. 2015). We acknowledge that LMICs is a comprehensive category and a variety of different setting fall indeed into it, so such heterogeneity is not a surprise. Other sources of variation in reported rates could be due to discrepancies in training, local practices and level of experience of accouchers, differences in level of implementation of restrictive episiotomy policies into actual practice and poor reporting practices (Ho et al. 2010).

The main strengths of this systematic review lie in its rigorous methodology and that it provides a comprehensive representation of currently available data on the frequency of BPT in LMICs. A large number of international databases were searched and broad inclusion criteria were applied to allow a wide range of studies to be screened for inclusion, hence providing a thorough picture of BPT in LMICs. However, there were also a number of limitations that are mainly related to the nature of included primary studies that need to be taken into account when interpreting the findings. The scarcity of studies reporting BPT in community births means that the results of this study should not be generalised to those births happening outside medical facilities. While it would be important to ensure appropriate representation of community settings, we acknowledge that data collection from community births might be challenging due to limited resources, difficulty in accessing remote areas, and security concerns in some settings. Additionally, several studies were classified as having high risk of bias, which might have an impact on the accurate estimation of the frequency of BPT. In many cases, the high risk of bias derived from the fact that the study was not designed to investigate BPT, but instead BPT was a secondary outcome. Moreover, we found that the majority of the studies failed to provide an adequate definition of BPT. There was also incomplete characterisation of the population under investigation, with most studies providing data on the age of the women only, without adequately describing the population being investigated in terms of important parameters such as the women’s socio-demographics characteristics and ethnicity nor birth related characteristics, such as parity and birthweight. These limitations could have contributed to the high level of heterogeneity amongst included studies which is a potential limitation to the external validity of our estimates. The high heterogeneity found in the meta-analyses and the poor quality of the studies means that the pooled estimates of BPT found in this review might be biased. Even so, it is important to report these estimates and highlight the associated issues so that future research can improve reporting practices and more accurate estimates cab be published. Although this review was not able to determine the exact rate of BPT in LMICs, it highlights the importance of improving data collection and reporting of BPT in LMICs. As Silva et al. (2016) found, in the context of mortality rates, it is unlikely that a one-size-fits-all approach will successfully improve reporting of birth-related outcomes. It is our view that future research agendas should aim to improve the quality of reporting and support advances in management of BPT in LMICs by: (1) collaborate with local and national authorities to improve the quality of data available on BPT in medical facilities, and to increase data collection in community setting; (2) disseminate evidence on best practice and support to implementation; (3) in-depth study of setting specific factors, within LMICs that impact care for the perineum, and how management of BPT can be improved. Given the emotional and physical distress BPT has on mothers, BPT should be considered as a core outcome and routine systematic examination of the perineum following childbirth should be performed to reduce misdiagnosis.

## Conclusion

Significant degrees of BPT affect more than 70% of women having a vaginal birth in LMICs. In this review, we provide insight into how the topic has been approached by researchers, limitations of currently available data and suggestions for improvements. We recommend that there is an urgent need to explore reasons for and devise programmes to reduce the apparent higher rates of episiotomies in LMIC medical facilities. Moreover, it is crucial to unveil BPT rates and outcomes within community based births in LMICs. Both issues are critical in view of their impact on women’s short and long term health and the potential impact on a woman’s decision regarding place of birth. Finally, the need for better reporting practices and uniformity of classifications is essential to enable appropriate management of such trauma. We believe that these recommendations are essential to improve outcomes for women following BPT particularly in the LMIC low-resource settings with limited facilities for managing chronic conditions. We urge policymakers in LMICs to prioritise this area of maternity care for future research, training programmes and quality improvement work.

## Electronic supplementary material

Below is the link to the electronic supplementary material.

Supplementary material 1 (DOCX 20 KB)

Supplementary material 2 (DOCX 17 KB)

Supplementary material 3 (DOCX 15 KB)

Supplementary material 4 (DOCX 2595 KB)

Supplementary material 5 (DOCX 2145 KB)

Supplementary material 6 (DOCX 2608 KB)
