# Supplementary material for: Birth-Related Perineal Trauma in Low- and Middle-Income Countries: A Systematic Review and Meta-analysis
Source: Matern Child Health J. 2019 Mar 26;23(8):1048–70. doi: 10.1007/s10995-019-02732-5 (PMC6606670; doi:10.1007/s10995-019-02732-5)
Supplement: Supplementary file 2 — Supplementary material 2 (DOCX 17 KB) [file 10995_2019_2732_MOESM2_ESM.docx]

Table 1- Data Extraction Form

| General Information | | | | | |
| --- | --- | --- | --- | --- | --- |
| 1. Country | | |  | | |
| 2. Date of Publication | | |  | | |
| 3. Study Period | | |  | | |
| Characteristics of the study | | | | | |
| 4. Study Design *(Cross-sectional, Case-control/case-series, cohort/longitudinal, CT, Incidence/Prevalence survey, unknown, other)* | | |  | | |
| 5. Sampling *(describe method of sampling)* | | |  | | |
| 6. Data source (*Medical records, special survey, interview, multiple sources, clinical data collected for the study)* | | |  | | |
| 7. Lower unit of data *(Cluster, individual, other)* | | |  | | |
| 8. Number of eligible subjects | | |  | | |
| 9.Population Studied *(Urban ,Rural, Mixed, unknown)* | | |  | | |
| 10. Socio-economic characteristics of the population | | |  | | |
| 11. Health state of the population | | |  | | |
| 12. Proportion of subjects lost to follow-up | | |  | | |
| 13. Characteristics of subjects lost to follow-up | | |  | | |
| 14. Setting *(National, Province/Region, City, Other)* | | |  | | |
| 15. Place of delivery *(home, hospital, mixed, other)* | | |  | | |
| 16. Forms of reporting data *(Crude, Standardised by population, both)* | | |  | | |
| 17. Are there data regarding risk factors? *(Y or N)* | | |  | | |
| 18. Comments | | |  | | |
| 19Frequeny | | |  | | |
| **Type of trauma** | Nº of cases | Denominator | | Percentage | Confidence Interval |
| **2nd degree** |  |  | |  |  |
| **OASIS** |  |  | |  |  |
| **Episiotomy** |  |  | |  |  |
