# Supplementary material for: Birth-Related Perineal Trauma in Low- and Middle-Income Countries: A Systematic Review and Meta-analysis
Source: Matern Child Health J. 2019 Mar 26;23(8):1048–70. doi: 10.1007/s10995-019-02732-5 (PMC6606670; doi:10.1007/s10995-019-02732-5)
Supplement: Supplementary file 3 — Supplementary material 3 (DOCX 15 KB) [file 10995_2019_2732_MOESM3_ESM.docx]

# Table 2 - Quality assessment parameters

|  | Adequate/ Inadequate/ Unclear |
| --- | --- |
| **STUDY ID:** | |
| Defines perineal trauma and distinguishes its degrees |  |
| Uses a random sample or whole population? |  |
| Adequate sample size ( >200 subjects) |  |
| Data reported as actual counts (rather than estimates) |  |
| Reports response rate and attrition/excluded |  |
| Confidence intervals, subgroup analysis |  |
| Study subjects described satisfactory |  |
