# Supplementary material for: Birth-Related Perineal Trauma in Low- and Middle-Income Countries: A Systematic Review and Meta-analysis
Source: Matern Child Health J. 2019 Mar 26;23(8):1048–70. doi: 10.1007/s10995-019-02732-5 (PMC6606670; doi:10.1007/s10995-019-02732-5)
Supplement: Supplementary file 4 — Supplementary material 4 (DOCX 2595 KB) [file 10995_2019_2732_MOESM4_ESM.docx]

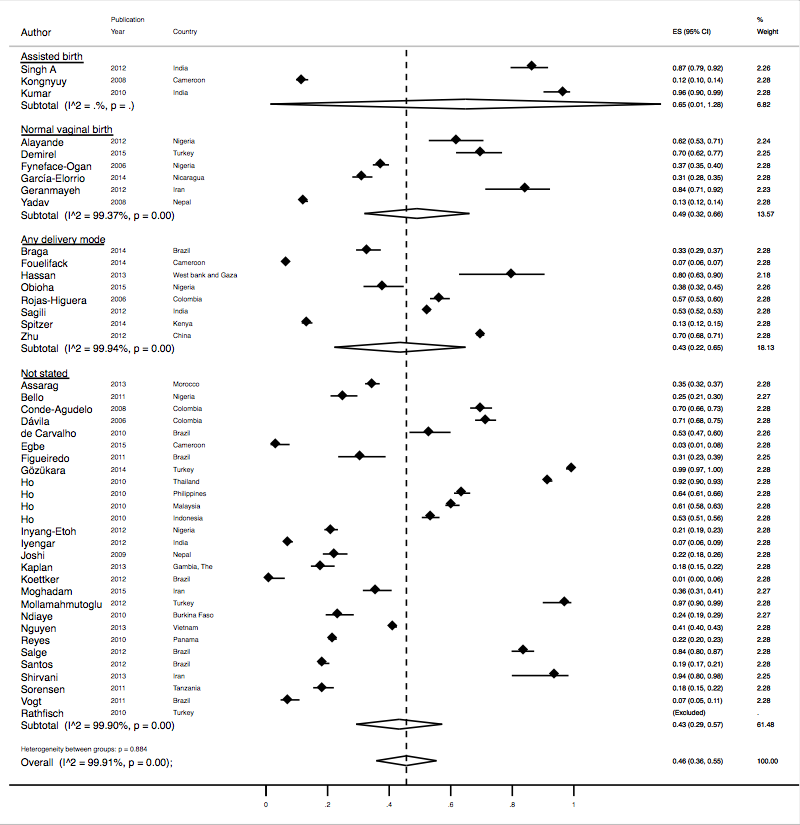


Figure 1 – Forest PLOT SHOWING RESULTS FROM META-ANALYSIS OF THE FREQUENCY of Episiotomy by MODE OF delivery. NS – Mode od delivery not stated in the study
