# Supplementary material for: Birth-Related Perineal Trauma in Low- and Middle-Income Countries: A Systematic Review and Meta-analysis
Source: Matern Child Health J. 2019 Mar 26;23(8):1048–70. doi: 10.1007/s10995-019-02732-5 (PMC6606670; doi:10.1007/s10995-019-02732-5)
Supplement: Supplementary file 6 — Supplementary material 6 (DOCX 2608 KB) [file 10995_2019_2732_MOESM6_ESM.docx]

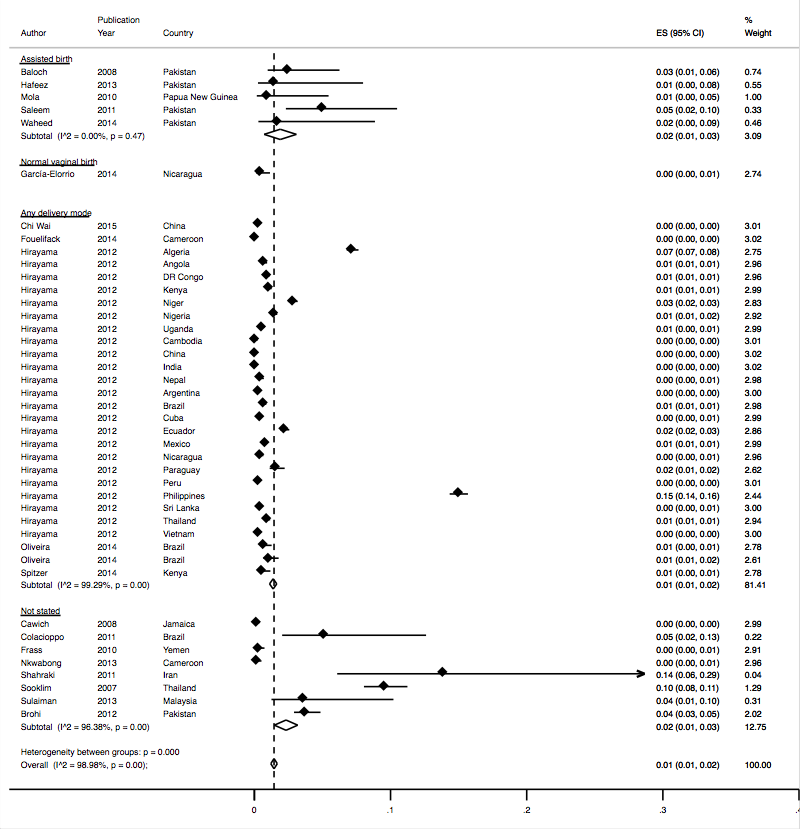


Figure 3 - Forest PLOT SHOWING RESULTS FROM META-ANALYSIS OF THE FREQUENCY of OASIS by MODE OF delivery. NS – Mode od delivery not stated in the study
